# UniDA: Uniform Device Access Framework for Human Interaction Environments

**DOI:** 10.3390/s111009361

**Published:** 2011-09-29

**Authors:** Gervasio Varela, Alejandro Paz-Lopez, Jose Antonio Becerra, Santiago Vazquez-Rodriguez, Richard José Duro

**Affiliations:** Integrated Group for Engineering Research, University of A Coruña, Ferrol 15011, Spain; E-Mails: gervasio.varela@udc.es (G.V.); alpaz@udc.es (A.P.-L.); jose.antonio.becerra.permuy@udc.es (J.A.B.); svr@udc.es (S.V.-R.)

**Keywords:** ubiquitous computing, distributed sensing, middleware, model and integration

## Abstract

Human interaction environments (HIE) must be understood as any place where people carry out their daily life, including their work, family life, leisure and social life, interacting with technology to enhance or facilitate the experience. The integration of technology in these environments has been achieved in a disorderly and incompatible way, with devices operating in isolated islands with artificial edges delimited by the manufacturers. In this paper we are presenting the UniDA framework, an integral solution for the development of systems that require the integration and interoperation of devices and technologies in HIEs. It provides developers and installers with a uniform conceptual framework capable of modelling an HIE, together with a set of libraries, tools and devices to build distributed instrumentation networks with support for transparent integration of other technologies. A series of use case examples and a comparison to many of the existing technologies in the field has been included in order to show the benefits of using UniDA.

## 1. Introduction

We are interested in the development of ubiquitous technologies for human interaction environments (HIEs). These environments must be understood as any place where people make their daily life, including their work, family life, leisure and social life. Therefore HIEs can involve different physical places such as the workplace, the home, the car and public places such as malls or sport centers, among others. In this context, hardware technology has evolved to the point where ubiquitous computing can stop being a dream of scientists and become a reality. Unfortunately, software and network technology, burdened by complexity and interoperability issues, is not at the same level yet.

During the last decade, computers have made the jump from the desktop to the kitchen, the living room, the car and the pockets of their users. Computers are now integrated in everyday objects, like appliances and consumer electronic products, just like Weisser envisioned in the early nineties [1]. Computers themselves are not the products anymore; they are simple parts of other products with specific purposes. They are not an end anymore, they are enablers for the development of services and products, in the same way electrical motors are used in multitude of products, like vacuum cleaners, fans, CD drives, without the user noticing their presence.

Regrettably, this integration of technology in our daily life has taken place in a disorderly and incompatible way. Many manufacturers have developed their own proprietary software, protocols and interfaces, and even when open protocols and interfaces have been adopted, their implementations have not been as compatible as they should be.

This has led to a situation where many competing technologies are fighting for the instrumentation market, each one of them incompatible with the others, with its strengths and weaknesses, and none capable of covering the whole range of needs of a HIE. Therefore, current HIEs are populated by devices that operate in isolated islands with artificial edges delimited by manufacturers. Entertainment devices in the living room, domotic networks for lighting, central heating and security, appliances in the kitchen, Bluetooth and proprietary devices in the car, *etc.*, every one of them operating on its own, without being aware of the state of the others or the user himself. This isolation is holding back the development of rich services that could result from the integration of multiple devices and information sources. Not to mention the waste in resources, due to the excessive computing power used and the inability to autonomously manage the power state of the devices.

During the last few years, largely motivated by the ubiquitous computing dream and hardware evolution, numerous technologies for device interoperation and integration have been proposed by industrial and academic institutions with varying success. From general purpose solutions like Jini [2] or IEEE1451 [3,4] to standardization proposals like UPnP [5] or solutions coming from ubiquitous computing and ambient intelligence projects, like AMIGO [6] or SAIL [7].

In this paper we are present UniDA, a framework for the development of solutions that require integration and interoperation of devices and technologies in human interaction environments. It provides a complete solution for building low cost distributed instrumentation networks with support for transparent integration of other technologies, while at the same time including tools for developers to operate and manage the network and its devices.

The UniDA framework has three conceptual components: A common conceptual model for the description of an instrumentation network, a uniform paradigm for device access and a distributed operation protocol for the interaction between the different distributed elements of the system.

In this paper, together with the description of those conceptual components, we present an implementation of that conceptual framework as a distributed system made up of an implementation of the model and uniform device access paradigm as a Java library, a set of software and hardware gateway components that make up an instrumentation network with the devices connected to them, and a distributed operation protocol to allow the communications between the library and the devices.

This paper is organized as follows. Section 2 presents some existing work in the area of HIEs. Section 3 provides an overview of the UniDA framework. Sections 4 and 5 are dedicated to a detailed presentation of the design and implementation of the UniDA framework. Finally, Section 6 provides some use case examples of the framework, and Section 7 compares the UniDA framework to other well known HIE instrumentation technologies.

## 2. Related Work

Domotic technologies are currently the most commonly used solution for home automation and instrumentation. Their components are installed in the homes during the construction of the building as a substitute or complement to traditional systems like light switches, blind open/close systems, security surveillance systems, *etc.*

Domotic technologies often make use of dedicated and proprietary communication buses, requiring the use of proprietary devices that support them. This is the case of technologies like EIB/KNX [8] or Lonworks [9]. Others, like X10 [10], use the power lines of the home to connect devices to each other, but they also require special supported devices.

The operation of these technologies often follows a P2P paradigm in which state changes from a device can be associated to state changes in other devices by grouping them together in a distributed way. When a device state changes, it sends a message notifying it, and the other devices grouped to that state also change their state. This way of operating is very effective for simple devices and simple configurations, but it can be a problem when managing large systems. Furthermore it doesn’t support complex functionalities like the ones available, for example, in multimedia devices.

Another typical problem of these technologies is that, in general, the configuration of the system must be set up by professional staff, due to the complexity of the technology and because they don’t provide configuration tools designed for the end users. Consequently, the functionality is static, and doesn't evolve or adapt to user needs and preferences autonomously or even manually.

The IEEE1451 [3,4] family of standards was an industrial attempt to address the problems of distributed sensing as a whole. This family is divided into different standards, each one of them aimed at solving a particular set of problems. The IEEE1451.2 and higher standards (IEEE1451.X from now on), concentrate on the definition of standardized physical interfaces for connecting sensors and actuators to microcontrollers. IEEE1451.1, on the other hand, is devoted to the problem of developing sensing applications. It proposes a software model that abstracts the typical features of a sensing/actuation node in a network. It leads to the definition of a series of software components that build a development framework for any type of application that requires distributed sensing/actuation capabilities. IEEE1451.0 is in charge of filling in the gap between the .1 and .X standards, defining an API that allows applications developed over IEEE1451.1 to transparently use the IEEE1451.X standards.

This framework provides a large degree of versatility in the development of applications. However, this same characteristic introduces several consequences in the final implementations. First, such a generic view of sensing/actuation technology leads to a very high level of complexity, and this makes its implementation difficult in hardware systems with very limited computational power. And second, as it is a very generic paradigm, it leaves the responsibility of defining the network protocols and the interfaces of its services to the vendors, leading to possible interoperability issues among products from different vendors.

Moving away from the installations automation world, Jini [2], presented in 1999 by SUN Microsystems, was one of the first commercial proposals for distributed device operation. Jini is a technology intended to alleviate the difficulties found while developing applications that make use of a multitude of distributed devices. Unfortunately, Jini has apparently fallen short in its efforts. It provides a distributed computing environment where software components (services) can announce their presence, and other software components (clients) can call them to perform tasks, but Jini does this in a very generic way, providing the developers with only the basic capabilities required to manage communications in a flexible and reliable way. Unfortunately, this leaves too much complexity in the hands of device and application developers, that must agree on which services and objects must be used to interoperate. Furthermore, Jini heavily depends on Java technology, so it is mandatory to run a Java Virtual Machine (JVM) to use it, which can be a problem when we are talking about integrating very small and cheap devices in the network, devices that would not be able to run a JVM while preserving their size and cost.

These characteristics have resulted in Jini not being widely used in the interoperability of devices or ubiquitous computing solutions. Nevertheless, Jini has been, and is, widely used for the development of *ad hoc* distributed systems, especially in the area of enterprise system monitoring or high-performance computing.

Another promising technology, and possibly the most heavily used one nowadays, is UPnP [5], a technology developed by a consortium founded and led by Microsoft. It is similar to Jini in purpose, providing devices with mechanisms to initialize its network stack appropriately, announce their presence in the network, discover other devices in the network and access the services that those devices provide. The main purpose of UPnP is to eliminate the device drivers, replacing them with common protocols that support the functionality of each type of device.

The main differences between Jini and UPnP are technological ones, thus, while Jini relies on Java serialization and its own protocols for communications, UPnP relies on standard protocols like XML based protocols, such as SOAP [11], on top of HTTP. Another key difference is that UPnP does not require the use of a JVM, so it is possible to use it in hardware platforms that do not provide Java support. This is a key characteristic of UPnP, and, probably, one of the reasons for its success as compared to Jini. UPnP has been widely used by the consumer entertainment devices industry, especially in multimedia network devices.

Unfortunately, UPnP has not achieved a huge general success. This is in part due to misuse of the technology. While many products advertise UPnP support, the reality is that they are implementing their own incompatible protocols on top of UPnP, rendering it useless from a compatibility point of view. Another problem is the hardware requirements of UPnP. As it relies on XML for communications, it is difficult to use it in devices with very low memory and processing capabilities without increasing the costs.

InterPlay [12], a technology developed by Samsung, is another attempt in the search for device integration in the home. InterPlay is very different to other technologies, like Jini, UPnP or the IEEE1454 standard, because it is not intended to be deployed on the final devices themselves, but on the devices were a final application will be in execution. In fact, it does not even provide a mechanism to communicate devices, it relies on existing technologies like UPnP or Jini for that, and uses plug-ins to support those different technologies.

InterPlay acts at a higher level than device interoperability technologies, providing high-level services to user applications. It presents a layered design with three layers. The first one is in charge of device interoperability; it contains the plug-ins to support the different device networking technologies, like UPnP, hiding device heterogeneity from the upper layers. The second layer provides device abstraction in a technology independent format, as well as aggregation of devices, content and user preferences. It is in charge of collecting information from all the available devices, and provides a unified vision of them. Finally, the third layer provides session management for applications, offering capabilities to manage the collaboration between multiple devices and access the devices and content made available by the second layer.

Ambient Intelligence (AmI) [13] and ubiquitous computing are research areas that, by nature, have to tackle the device interoperability problem. AmI applications must use and manage a wide range of devices to do its job. Because of this, AmI and ubiquitous computing related projects like AMIGO, PERSONA or AmI-Space have also developed solutions for device interoperability.

The AMIGO project [6,14] uses standardized solutions as much as possible, developing abstractions to hide the heterogeneity of the devices and translate their functionality to AMIGO compatible services. AMIGO proposes UPnP as a standard for hardware access so, when a non-UPnP compatible device needs to be used, it is necessary to develop proxy services that use *ad hoc* drivers to interact with the device and translate its interface. They have developed proxies for two widely used domotic technologies, BDF and EIB.

The AmI-Space project [15] proposes a similar solution to AMIGO, integrating technologies through the use of encapsulating proxies. The main difference is that they also provide a hardware solution to simplify the physical integration of technologies as well as the development of new devices.

The PERSONA project [16] uses SAIL [7], a sensing abstraction layer designed to provide access to wireless sensor networks (WSN). SAIL uses a layered design in which a first layer contains the logic to directly interact with the different WSNs supported, a second layer that abstracts the functionalities of the WSNs as OSGi services, and a third layer that exports those services with interfaces that are compatible with external software, like UPnP or in the case of PERSONA, an event based system.

Another interesting approach for home device interoperability is the one adopted by some research projects that are building Intelligent Domotic Environments. A typical approach to build such environments is to develop a domotic control gateway. This is a device that is connected to domotic buses, and is in charge of providing homogeneous access to those technologies and, in some cases; some extent of high level intelligent behaviour.

A prominent example of these approaches is the Domotic OSGi Gateway (DOG) [17]. They are developing a software Home Gateway that can be installed on an embedded PC and contains plug-ins to support existing domotic technologies (currently KNX and OpenWebNet). This PC can be connected to a home network and provide a homogeneous API to access the available domotic devices. This is achieved by using different specific domotic technology plug-ins, with network gateways to connect the network to the domotic buses. DOG also provides high-level functionalities on top of the basic device interoperability layer. It makes use of the DogOnt [18] ontology as a model for a home automation environment and its devices. This use of semantic web technologies to model the environment provides the system with inference capabilities, allowing operations like device abstraction leveraging the hierarchy of classes, command validation at runtime, runtime knowledge of the commands a device can receive, introspection of device capabilities at runtime, *etc.*

Finally, some interesting developments can be found in the improvement of interoperability among domotic technologies. A prominent example of this approach is DomoNet [19], where the interoperability of domotic technologies is improved by means of a virtualization of heterogeneous domotic networks. The main characteristic of this solution is that, by using custom adapter software for each technology, applications developed with libraries for particular domotic technologies can use devices from other technologies. Another example is [20], where a fuzzy logic control system for domotic environments is introduced. They use a fuzzy markup language to model a generic domotic network and fuzzy logic to define its behaviour independently of the concrete technologies involved. This behaviour is finally implemented by a distributed multiagent system.

From the paragraphs above, it is clear that there are a lot of technologies fighting for the HIE instrumentation market. The main difference between them and the UniDA framework is that UniDA addresses the problem as a whole, providing an integral solution for the instrumentation of HIEs allowing the building, management and operation of device networks and the development of applications that exploit those networks.

Furthermore, UniDA differs from existing technologies in many critical ways. With respect to Jini, UPnP and AMIGO, UniDA offers a more resource efficient solution, allowing for the direct integration of low power and legacy hardware devices. It also offers a specific solution for home/office instrumentation, so it eludes the risks of letting the definition of software services interfaces in the hands of the final manufacturers. Additionally, is important to note, that, as will be illustrated in this paper, it includes support for the transparent integration of other technologies, like domotic technologies or even Jini or UPnP.

Compared with Interplay, SAIL and the home gateway approach, which are very similar in nature. UniDA has prominent differences in its approach. Interplay, SAIL and the home gateway completely hide the devices behind software layers, allowing the utilization of multiple hardware technologies from a single point, but not trying to provide an alternative to those technologies. We think that the best way to go is to take intelligence to the end devices as much as possible, letting the upper levels of the system be only their controllers or monitoring agents. Thus, UniDA does not hide the fact that there is a network of devices operating in a distributed manner.

UniDA shares some important similarities with AmI-Space and the DomoNet approach. All provide software components to enable the interoperation between existing technologies, even using proxy like components for the integration of devices. Nevertheless, on the one hand, DomoNet is focused on interoperability of existing technologies, while UniDA provides a solution for building new instrumentation networks integrating those technologies. On the other hand, AmI-Space does not provide a common model of the instrumentation network, nor a uniform paradigm to operate the different devices.

## 3. UniDA Framework Overview

The UniDA framework is a solution for the integration and interoperation of devices in Human Interaction Environments. It is possible to use UniDA for two different, but interrelated, purposes. As an abstraction layer it allows the development of applications that handle hardware devices with independence of the technologies used in each device and their particular characteristics. As a complete HIE instrumentation solution it permits building distributed device networks with support for the transparent integration of existing installations and technologies.

**Figure 1.**
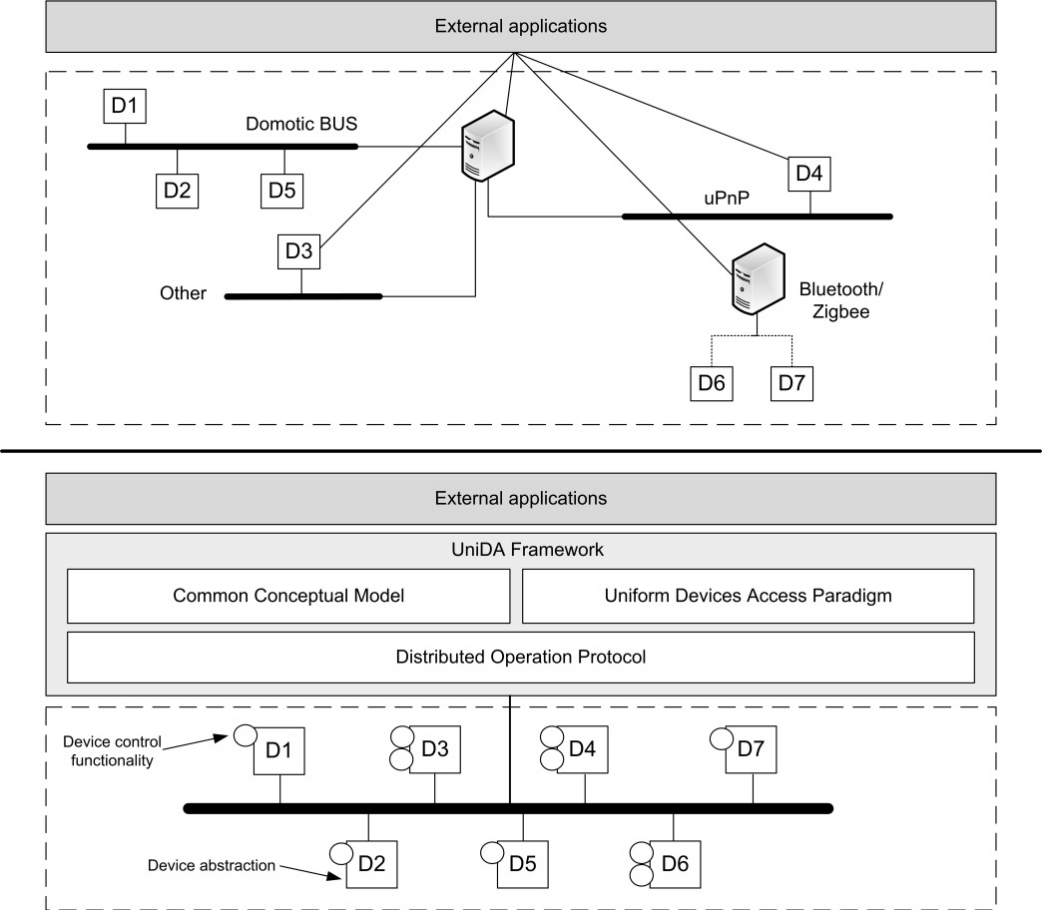
High-level architecture of the UniDA framework.

Figure 1 compares the vision of the hardware available in a HIE from applications that interact directly with the devices, to the vision of applications that use the UniDA framework to interact with the same devices.

In the first case, the applications have a heterogeneous vision of the network of devices; they must include particular logic to interact with each specific technology and device available in the network, thus complicating the development process and making the addition of new devices more difficult, as they need to take care of all the complexities and particularities of each hardware technology deployed in the installation. In the second case, the applications have a homogeneous vision of the network, they are able to use the same concepts and operations to interact with every device, independently of their underlying technologies. This way they do not require knowledge of specific technologies, they can use devices from different technologies homogeneously, and new devices can be easily added to the network without requiring any modification of the application logic.

There are two points of view to describe the UniDA framework. On one hand there is a set of abstract conceptual components that made up a conceptual framework for the development of solutions in the HIE instrumentation field. On the other there are a set of elements that implement this conceptual framework, providing usable solutions for the design, implementation and deployment of HIE instrumentation systems.

The conceptual framework is made up of three components. A common conceptual model for the description of an instrumentation network and its devices, a uniform paradigm to model the interaction with devices, and a distributed operation protocol for the interaction between the different distributed elements of the system. The conceptual framework will be described in detail in Section 4 of this paper.

These components are realized by two main elements, complemented with some configuration tools, which allow developers to use the model to interact with the available hardware devices within their software:

- A software library (developed in JAVA) implements the common conceptual model and provides a simplified façade with the operations supported by the uniform device access paradigm. An ontology [18] is used to support the model and enrich its semantics, allowing some useful inference capabilities, like device class inheritance.
- Proxy-like components, called device gateways, one for each supported instrumentation technology, are in charge of translating the abstract concepts managed by UniDA to specific concepts of a particular technology. These gateways are usually deployed on remotely accessible embedded hardware devices that are physically connected to the end devices or to other instrumentation network technologies.

The library and the device gateways will be further discussed in the Section 5 of this paper. The UniDA conceptual framework, together with its implementation components builds a complete framework for interoperability of instrumentation hardware, alleviating the development costs of applications and installations for such heterogeneous environments as HIEs are.

## 4. UniDA Conceptual Framework

As presented in the overview of Section 3, the UniDA framework is constructed around three conceptual components. These components make up a complete conceptual framework for the development of instrumentation systems for HIEs. Therefore, when using UniDA, these components represent the shared knowledge that developers, installers, and even manufacturers, need in order to design and implement applications, systems and devices for HIEs. The next section is devoted to the description of these three conceptual components.

### 4.1. Common Conceptual Model

In UniDA, the homogeneous vision of a heterogeneous network of technologies is provided by a common conceptual model with a uniform device access paradigm. This common conceptual model takes the similarities between the different existing technologies, and builds a new set of concepts that represent the essential characteristics of every instrumentation technology. This set of concepts abstracts the peculiarities of a heterogeneous instrumentation network, providing developers with a homogeneous language to interact with devices, backed up by a set of software and hardware components that translate it into the particular concepts and technologies required to interact with specific devices.

When using UniDA, developers, and more specifically, the applications that must make use of the instrumentation network, only need to talk in the common language provided by the common conceptual model. Therefore, this conceptual model must be, on one hand, powerful enough to support all the characteristics and functionalities required by the applications, and on the other, simple enough to be easy to use and not transfer the hardware complexities to the applications.

Trying to hide all the particularities and complexities of every instrumentation technology is not an easy goal. Even if a set of common concepts can be easily identified, the wide variety of devices and characteristics available can pollute the model with an increasing number of low-level concepts such as the types of devices that could exist, the different states a device can have, the different operations supported, *etc.*, complicating the usage of the model with a lot of unneeded details and information. It would be really difficult and costly to try to identify in advance every type of state or functionality that can be available, and in fact, it must be taken into account that new types of devices may appear in the future. Consequently, it was decided to decouple the model into two models, a simple abstract model to represent the high-level concepts and relationships that support the operation and management of an instrumentation network, and another model to act as a taxonomy of the specific instances of those concepts and the relationships that could exist. This way, the framework can easily accommodate new types of devices, by only defining them in the taxonomy using the simple abstract model, and every operation supported by the framework can be performed on top of those abstract concepts, isolated from the particularities of each device.

One of the best ways to describe such a taxonomy is to use ontology description languages [21,22], like RDF [23] or OWL [24]. These technologies have been widely used in recent years due to their adoption as key technologies for the semantic web. They provide powerful mechanisms to describe comprehensive semantics about concepts, content and relationships between them. This, along with ways to exploit those relationships by inference engines, provides powerful tools to reason with the concepts supported by the ontology. Another interesting point about ontologies is that they can be a highly isolated piece of software, so they can be easily shared between different software projects, with the added benefit that if two software projects use the same ontology to model some environment, they can interoperate very easily, and improvements to the ontology are reflected as direct improvements in every software component using it.

There are some device description ontologies available, unfortunately many of them are too related to computer devices [25,26] and, in general, they are only conceptual ontologies, and it is difficult to find publically accessible and usable implementations of them [21,27]. Nevertheless, one prominent example of a device ontology is the DogOnt ontology [18], a device reasoning ontology developed by the e-Lite research group of the Politecnico di Torino, in Italy.

The DogOnt ontology is a very comprehensive taxonomy of devices typically used in home environments. It was developed as a part of the Domotic OSGi Gateway (DOG) project. DOG is a software home gateway that can be installed on an embedded PC and contains plugins to support current existing domotic technologies, providing a homogeneous API to access the available domotic devices. The DogOnt ontology serves as the model of a home automation environment for the DOG gateway and its client applications.

We decided to use the DogOnt ontology as our taxonomy of devices, not only because it is very complete, well supported and updated, but also because it matched very well the requirements of the conceptual model that we were designing. Our model is based in three main concepts: the devices, the functionality that those devices provide and some kind of proxies or gateways that connect the devices to the instrumentation network. The DogOnt ontology offers very good support for these concepts, the device concept is directly supported and its functionalities are represented by two elements, control functionalities and notification functionalities. Furthermore, even the devices proxy concept is directly supported by a gateway concept represented in the taxonomy as ‘DomoticNetworkComponent’.

**Figure 2.**
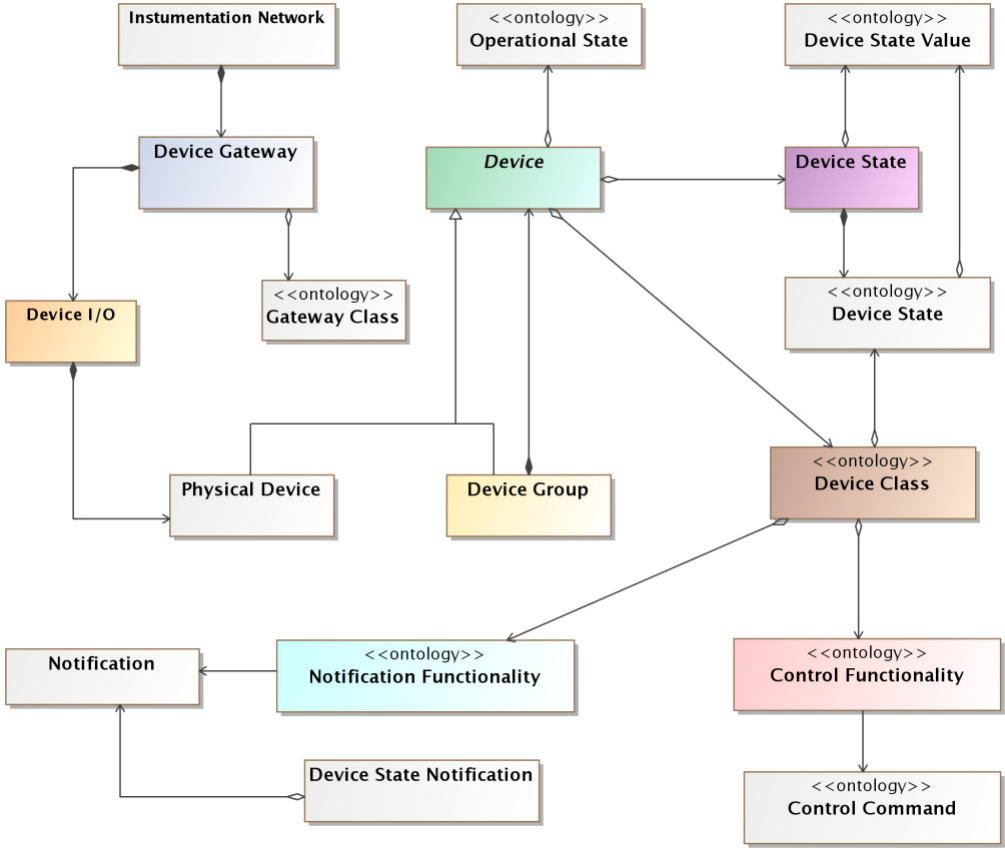
Simplified vision of the proposed conceptual model for instrumentation networks.

Therefore, many of the concepts found in the model presented here are adapted, and even some of them directly extracted, from the DogOnt ontology. Thus, the conceptual model, as shown in Figure 2, is made up of eight main concepts:

- **Devices**. These are the devices that a user expects in his instrumentation network. They provide end users with services and functionalities. There exist two different kinds of devices; physical devices and groups of devices.
- **Physical devices**. They are a conceptual representation of the real hardware devices.
- **Groups of devices**. Multiple devices can be grouped together, and a group has the same entity as one device. Thus, a user or an application can send commands, queries, *etc.* to a group of devices in the same way (transparently) as to a single device.
- **Device gateways.** They are the connection point between the devices and the instrumentation network. They act as a control point for the devices, controlling them and transferring to them the commands that came from the instrumentation network, and *vice versa*.
- **Device I/O**. Physical devices are not directly connected to the gateways, they are connected through what we call a device I/O. A gateway has a set of device I/Os to which devices can be connected (this set can be static, or dynamic) and each device I/O has a list of states that restrict the type of devices that it can hold, so the I/Os of a gateway specify the number and type of devices it supports.
- **Classes of devices**. The class of a device represents its type. It supports inheritance thanks to the use of ontologies.
- **Device states.** They represent the type of states that a device has. A device can have multiple different states and they are specified by its device class.
- **Device commands** and **device notifications**. Together they represent the functionality of a device. Commands can be received by a device and it usually reacts by changing its state and acting over the environment changing it. Notifications represent changes in the state of a device. Devices send notifications to notify the clients of the instrumentation network about a change in its state.

As the objectives of this proposal are a bit different and broader than the DOG objectives, even if some concepts of the model presented here directly matches concepts from the DogOnt ontology, there exist important conceptual differences. One of the more prominent differences is the inclusion of the device I/O concept. Even though it is a concept that developers do not need to know, it is very important for the internal workings of UniDA and for the configuration of the system. There are gateways that are able to automatically detect when a device is connected to them and identify it. These gateways support a variable number of devices and make use of dynamic device I/Os. Thus, when a device is connected to them, they automatically create a new device I/O for the device and configure it. Furthermore, there are gateways, known as static gateways, that have a fixed number of physical interfaces to connect passive devices and, in order to be compatible with very simple or legacy devices, they are usually unable to detect the presence of the device, requiring manual configuration.

This last case is one of the main reasons for the introduction of the device I/O concept. It provides the system and installers a way to know what kind of devices can be connected to a particular gateway. Therefore, when a static gateway is incorporated to the system, the installer needs to manually configure the device I/Os the gateway is reporting. To do that, he specifies the class of devices that are connected to each device I/O. It must be stressed that not every device can be specified as connected to a particular device I/O, due to the fact that each one of them has an associated list of states that it supports. For example, a digital I/O will only have associated an on/off state to it, so that, only device classes that support that state may be associated to that device I/O. This configuration can be carried out with a web based configuration tool available for system installers.

Another important key aspect of the proposed model is that it was decided to make use of the DogOnt ontology only as a taxonomy of metadata for the concepts considered here. That is, an implementation of the proposed conceptual model doesn't have to populate an ontology with instances in order to represent the proposed concepts. It can represent the concepts with any technology, like object-oriented programing, and only use the ontology as a repository of metadata about the different concepts managed by the system.

### 4.2. Uniform Device Access Paradigm

The common conceptual model will not be complete without a description of how these concepts can be operated to interact with the instrumentation network, this description is the uniform device access paradigm. It provides a set of generic operations that can be used to manage and command the different elements that populate the system, allowing client applications and other elements to access the functionality supplied by the available devices.

The uniform device access paradigm is made up of a very small set of operations that, by relying on the common conceptual model, are sufficient to access any device functionality defined in the ontology. It has three main device access operations complemented by a set of management ones:

- **Query a state of a device**. Every state that a device supports can be queried at any moment.
- **Send a command for execution**. It is possible to send commands to the devices. The commands supported by a device are defined in its metadata, that is, specified in the DogOnt ontology for every device class. Once a device receives a supported command, it must act in accordance to the semantics of the command.
- **Subscribe to a device state**. Devices must send notifications when one of their states changes. Therefore, clients can subscribe to those notifications in order to receive them.

These three operations are complemented with a set of management operations that will allow clients of the model (developers and applications) to find the devices and gateways available or defined in a particular instrumentation network, as well as access their descriptive information and metadata.

Therefore, the concepts provided by the common conceptual model and how to operate them (the uniform device access paradigm) is the only knowledge of the instrumentation network that application developers need to have, as any implementation of UniDA will provide them with an API to manage those concepts and issue operations, completely decoupling the application logic from the particular hardware technologies used to build the instrumentation network.

Finally, in the next section we describe the third component of the UniDA framework: the distributed operation protocol that enables the interaction between the different distributed physical elements of the system.

### 4.3. Distributed Operation Protocol

Due to the characteristics of HIEs and their intrinsic ubiquitous nature, devices need to be physically distributed throughout the environment in order to monitor and act over it. UniDA framework is designed to support the distributed operation of some of its elements, in particular, the elements that implement the device gateway concept can be installed distributed to build a network of devices.

The objective of the distributed operation protocol is to allow the interaction between the clients of the framework (applications) and the devices deployed throughout the environment. The protocol must support all the operational, maintenance and management tasks required for the correct operation of the instrumentation network. These include support for:

- **Control operations**: query states, send commands, subscribe to state change notifications.
- **Management operations**: access to descriptive information about devices and gateways, discovery of devices/gateways available in the network, announcements of new devices/gateways available.
- **Maintenance operations**: Detection/notification of failures, announcements of device disconnections.

This kind of protocol can be modelled and implemented in multiple ways, being a very good option to use standard protocols. For example, the operational functions could be easily modelled by means of a RPC (Remote Procedure Call) protocol, like CORBA, XML-RPC or SOAP [11,28,29]. For the discovery of devices there are also good examples of standard protocols that could be applied, like SLP, SSDP or UPnP.

Unfortunately, these protocols are large and complex [11,28], they use text based communications through the HTTP protocol, and they use the XML language to format the content of the messages [5,11,29]. As the logic required to control and command many of the devices available in a HIE will be fairly simple, using this type of protocols could cause that, in many cases, the power and processing time required for the communications logic be even larger than that required for the functionality logic. These protocols would hinder the construction of low cost, low power and small size hardware gateways for simple devices, like the one we are developing as part of the UniDA framework, (see Section 5.3 for more details).

There are two ways of coping with this. On one hand, one could use two different communications protocols. Standard protocols to interact with high end device gateways and an *ad hoc* protocol to interact with low end/low cost device gateways that command simple devices. On the other, one could use a single *ad hoc* protocol for all the device gateways.

The first approach has the problem that the instrumentation network would be made up of two different types of device gateways that cannot interact directly between them. Thus it would be more difficult and inefficient to implement autonomous distributed behaviour capabilities directly in the gateways, which is one of our future goals.

As a consequence a new protocol has been designed for the interaction between the components of the instrumentation network. It is a simplified stateless message interchange protocol, based on the interchange of a series of predefined messages without requiring establishing sessions or connections. The reason to avoid a session based protocol is that a high percentage of the interactions present in an instrumentation network must be quick (kind of real time) and involve a very small amount of data transfer. Think for example of a request to power up a lamp or a notification of a state change in a door. A session based protocol would introduce a lot of overhead, and, in many cases, the overhead could even be larger than the communications act itself.

The communications protocol is defined as a set of messages that can be exchanged between the components of an instrumentation network. These messages are composed of three parts, a header, that contains metadata about the message, the content of the message and a checksum. Therefore, the protocol is defined in an abstract way and it can be implemented by using any connectionless transport protocol. In order to keep the hardware requirements for the communication logic as low as possible, it is mandatory to implement it by using a binary encoding for the messages instead of using text-based ones like XML. The proposed protocol contains thirteen different types of messages to support all the functionality required by the instrumentation network, like gateway discovery and announce, command execution or notification messages.

The interchange of messages is modelled as a request/response protocol, except for the gateway announce message and the notification messages. The announce message must be broadcasted to all the members of the instrumentation network, so everyone can know which gateways and devices are connected to the network. The notification messages must be multicasted to all the members of the network subscribed to them.

The announce messages do not have to be answered or acknowledged, but they must be sent periodically by every gateway, in order to alleviate possible reception problems, and because it is also used by other elements of the network to monitor the state of remote gateways and devices. The period of gateway announcements is variable, defined by each gateway according to its requirements, and it is notified to the monitoring elements using the gateway announce messages.

Notification messages can be acknowledged, but it is not mandatory depending on the implementation. Every other message has an associated response (or acknowledge) message, thus, it is mandatory for the implementation of the protocol to establish some kind of reception control mechanism in order to guarantee the reception of the messages, or at least, to be able to detect communications errors and act accordingly.

## 5. UniDA Library and Gateways

In the previous sections we have presented UniDA as three conceptual components, a common conceptual model of an instrumentation network, a uniform device access paradigm that models the interactions between the members of the instrumentation network and a distributed operation protocol for the remote interaction between some of the members of the network. In the present section we are going to show an actual implementation of those components.

**Figure 3.**
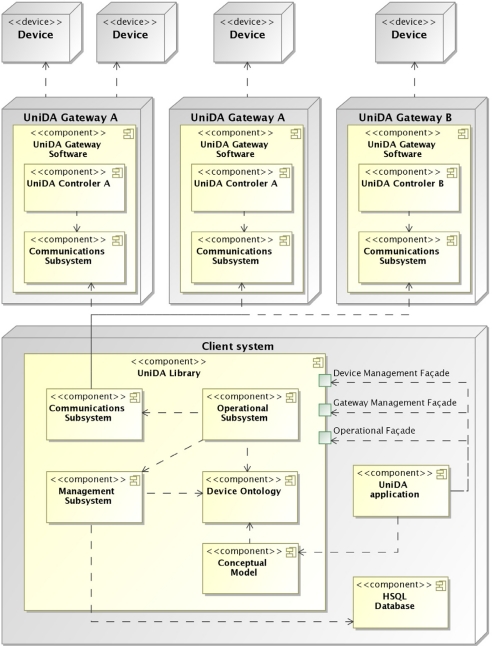
Architecture diagram of an UniDA system.

Figure 3 shows a diagram of the UniDA framework implementation architecture. As can be seen, it is divided into two main components, the UniDA Library and the UniDA gateway. The UniDA Library is an implementation of the common conceptual model, the uniform device access paradigm and the distributed operation protocol as a J2SE library. The library (and its dependencies) is the only software component that a client application needs in order to command and control an instrumentation network.

The UniDA Gateway is the realization of the Device Gateway concept defined in the common conceptual model. There can be multiple implementations of the UniDA Gateway, from generic implementations that will be run in common hardware, such as computers or smartphones, to embedded implementations for specifically designed gateways. It is in the gateways where the particular control logic of each device resides, and by using some components of the UniDA library (or implementing them for embedded gateways), like the distributed operation protocol, they provide access to devices to clients of the UniDA Library. That is, UniDA Gateways translate the common concepts managed by UniDA to the particular ones used by each device technology.

### 5.1. UniDA Library

The functionality of the library is presented to external clients through a reduced set of concepts (classes of objects, as it is implemented using JAVA, an object-oriented language) directly implemented from the proposed conceptual model shown in Section 4, and three façades that export the operations to manipulate those concepts in a uniform way. There exists one façade for the operational functionality of the instrumentation network, like query the state of a device or issue a command to a device, and two façades for management operations, one for device management operations and another for device gateway management operations.

As can be seen in Figure 3, the library is implemented as five modules that are encapsulated behind the three façades: an implementation of the common conceptual model; a management subsystem, in charge of providing access to information about the devices available in an instrumentation network; an operational subsystem, in charge of providing control capabilities over those devices, a communications subsystem, that implements the distributed operation protocol; and a device ontology module that manages the access to the DogOnt ontology. These modules will be described next, except for the common conceptual model implementation, which is only an object-oriented implementation of the model proposed in Section 4.1.

#### 5.1.1. Management Subsystem

The management subsystem, implements two different sets of operations related to two different concepts. On one hand, it provides operations to create new devices or gateways, remove existing ones, edit their information and relationships, *etc.* On the other, it provides query functionalities to allow applications to access the information about the devices and device gateways available in an instrumentation network.

The management subsystem is designed to require minimal, or even no interaction with the devices. This is an important characteristic because one of our goals was to support the off-line management of the instrumentation network, so that, an installer could set up a network at his office and then deploy it in the physical installation with minimal intervention.

Therefore, the management subsystem does not require communications with the devices, any change in the installation is stored in a database. Once the installation is deployed and on-line, the operational subsystem interacts with the network, and uses the management subsystem to update the state of the devices and gateways to reflect the current state of the network.

The installation database contains the complete definition of a particular installation. This definition is a series of instances of the concepts defined in the common conceptual model, more specifically devices, device gateways and the relationships between them, the ontology and the physical environment.

As we wanted to make the device access library small and easy to use and deploy, so that even small standalone control applications could use it, we decided to reduce the deployment requirements by not using a classical database management system (RDMS) with a separate server; instead, we used an embedded RDMS, HSQLDB [30].

As shown in Figure 3, the management subsystem also makes use of the DOG ontology. As mentioned before in Section 4, the ontology is used as a taxonomy of the different types of devices and device gateways that can exist, as well as a source of semantic metadata about those concepts, like their relationships, their properties, *etc.* A complete description of how the ontology is used will be provided later in this paper.

#### 5.1.2. Operational Subsystem

In a similar way to the management subsystem, the operational subsystem implements two different sets of functionalities. On one hand, it implements the operations required to control the devices and the instrumentation network. On the other, it supports the maintenance operations by implementing the functionality required to continuously monitor, in real time, the operational state of the network and update the management database through the management subsystem in order to reflect the current state of the network.

The command functionalities are all implemented as asynchronous operations. We decided to take advantage of the fact that the protocol of the instrumentation network (Section 5.1.3) is designed as a connectionless protocol, and the control and command capabilities of the library were implemented in an asynchronous way, so that the client applications can operate the network without actively waiting for responses. This allows for an easier development of more responsive and efficient applications.

The command functionalities also contain the logic required to control groups of devices, so it is able to execute the same operations over multiple devices. Even if a device of the group is not available, it stores the operation in a queue and sends it to the devices as soon as it is available.

The monitoring of the device network is achieved by continuously receiving and processing gateway announce messages. They must be sent periodically by the gateways to maintain the management subsystem updated with the last known information about the network, like new devices that were connected to the network, devices or gateways that were disconnected, or failures detected.

#### 5.1.3. Communications Subsystem

The communications subsystem implements the distributed operation protocol independently from the other subsystems, and it is divided into two components. On one hand, a message handling system, made up of a set of messages, logic to code and decode them in a binary format, and logic to manage their processing, and on the other hand, a communications channel implementing the particular logic to send and receive messages from a specific network technology. The internal division of the communications subsystem into two components allows reusing the message system with different network technologies.

Following the guidelines for the implementation of the protocol described in Section 4.3, the messages are encoded as byte data in little-endian format. The selection of little-endian was made because many of the most commonly used low power microcontrollers available in the market, such as the Microchip PIC or the Atmel Atmega, internally represent data in little-endian, thus, the use of big-endian format, more of a network standard, would require a translation phase for every message. Bearing in mind the simplicity of the typical interactions in an instrumentation network, in gateways that use embedded hardware the translation process would be even more costly than the interaction itself.

In the current implementation of the UniDA Library, the message handling system uses one thread to continuously receive messages through the communications channel, these messages are decoded and stored in a message processing queue. This queue is processed by message handler objects containing the logic to process a message of a given type. They are the connection between the communications module and their external clients, the operational subsystem and the gateway controller, see Figure 3.

Bearing in mind the capabilities of low power microcontrollers and the sessionless requirement of the protocol, the default implementation of the communications channel uses the UDP transport protocol, and broadcast messages for discovery and notification messages.

As mentioned in Section 4.3, every message, except those used for gateway announce and notifications, has an associated response. This response is used by the protocol implementation to guarantee the reception of the message by its receiver, or at least, in the worst case, detect a communications error. A simple exponential backoff algorithm is used to resend the message *N* times if its response is not received during a certain period. If the maximum number of repetitions is exceeded, the message is cancelled and the component that requested the operation to the communications subsystem is notified of the error, so that it can take the appropriate actions. The number of resends and the expiration time of the first response can be specified independently for every operation requested to the communications subsystem.

The current UDP implementation sends messages as a single UDP datagram. It is part of the future work to modify the protocol to support multiple datagram messages, so that large messages can be used if required.

#### 5.1.4. Device Ontology

One usual problem of ontologies is that ontology management requires lots of memory and processing power to achieve low response times. As it was of paramount importance to keep the device access library light and responsive, it was decided to use ontologies in a limited way, that is, only as a repository of metadata, instead of using them directly for storing instances of the concepts and reasoning with them. Therefore, the UniDA Library uses the ontology exclusively as a taxonomy of the different types of devices and gateways that could exist in an installation, and as a repository of semantic metadata about those devices, their properties and their relationships.

A little module was designed and developed to manage the access to the ontology. This subsystem exports the ontology through a series of query methods that return metadata about the different concepts managed by the system. The biggest benefit of totally encapsulating the ontology behind this module is that the ontology can be updated or changed without affecting the other components of the system.

The ontology subsystem is implemented using the OWL-API [31] JAVA library for reading the DogOnt ontology, which is described in the OWL language, and the Pellet [32] library as the inference engine for the management of complex relationships between concepts. To reduce the hardware requirements and response times, we are using the basic query capabilities of the OWL-API whenever possible, and only relying on the inference capabilities of Pellet for traversing complex inheritance hierarchies. It is important to emphasize that the ontology module is only used by the UniDA Library, and it is not required by the gateways, which only need to know, as simple strings, the identifiers of the concepts related to the devices they manage, and their translations to the concepts used by each specific technology.

### 5.2. UniDA Gateways

In a UniDA based system the gateways are the elements in charge of translating the concepts from the common conceptual model and the uniform device access parading to the particular concepts and interfaces required by each specific device. Their job is to control hardware devices and to interconnect them building an instrumentation network that can be accessed and managed by the clients of the UniDA Library. Therefore, gateways are highly coupled to particular devices or technologies, and usually include specific software and hardware.

To this end, a UniDA gateway should include: The particular control logic for each device connected to it, that is, the device drivers. And an implementation of the distributed operation protocol so that it can interact with other elements of the network.

Depending on the requirements of the hardware devices, there can be many UniDA gateways deployed on very different hardware. For example, there can be gateways deployed on generic platforms, such as PCs, for devices that require complex control software, like domotic technologies; gateways deployed on smartphones to provide access to their sensing devices; or gateways deployed on custom embedded hardware to control simple devices or build new hardware devices directly compatible with UniDA.

**Figure 4.**
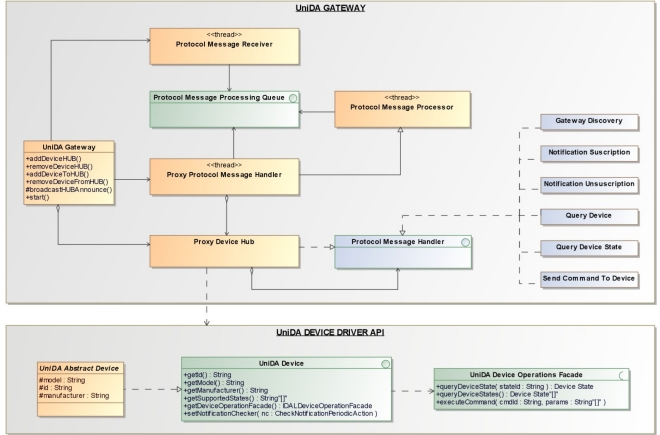
Simplified class diagram of the generic gateway software.

We have created a generic UniDA gateway software component, shown in Figure 4, which can be used to build new gateways. It is implemented in Java using the J2SE API so that it can run in any device with a J2SE virtual machine. It is a reference implementation of a UniDA gateway, with all the logic required to interact with the UniDA network and manage the processing of requests and notifications.

The generic UniDA gateway component is designed to simplify building new gateways. For this, developers need to provide the particular logic to control a device, that is, the device drivers. This is achieved by implementing two interfaces, the *UniDA Device* and the *UniDA Device Operations Façade* for each type of device. Finally it is also necessary to implement a main class to add the devices to the *UniDA Gateway* and start its execution.

Figure 4 shows a simplified class diagram of the generic UniDA gateway. The interfaces of the UniDA device driver API are those that developers must implement to develop new device drivers. The generic gateway groups devices into hubs, the *Proxy Device Hub*. Each hub is announced to the instrumentation network as a UniDA gateway, so one generic gateway can represent multiple UniDA gateways. This way, multiple UniDA gateways can be run in the same computer using fewer resources and sharing the same communications subsystem

Using the generic UniDA gateway component we have built gateways that allow the integration of some existing technologies into a UniDA instrumentation network. These gateways are an integral part of the UniDA framework, so they can be used by developers and installers to build their systems. By using these gateways, any application that uses the proposed framework will be directly compatible with the devices and technologies supported by these gateways, such as EIB/KNX domotic networks, UPnP media renderer devices, PJLink compatible projectors or Android smartphones.

### 5.3. UniDA-D

HIE applications present high requirements of contextual information about the environment and their users. The majority of this information comes from sensors that are deployed throughout the environment and they must be integrated within it in the most transparent way possible, that is, they must blend in. Consequently, the technology supporting them must allow them to be as small and cheap as possible.

The generic UniDA gateway component is useful for building UniDA gateways in the case of complex technologies or devices with high processing requirements whose hardware requirements are too high to transparently embed it in the environment with the sensors and actuators. Therefore, we have built a hardware gateway, called UniDA-D, designed to allow the embedding of sensor and actuator devices in an HIE and their connection to a UniDA instrumentation network. It also can be used as a reference platform for the development of new hardware devices directly compatible with UniDA that contain a gateway integrated within them.

**Figure 5.**
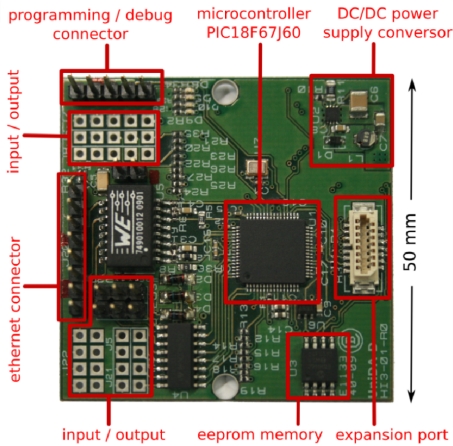
Picture of the prototype version of the UniDA-D hardware gateway.

UniDA-D is built with commercial off-the-shelf (COTS products) hardware to keep its costs low, and is designed to be small enough to fit in the standard connection boxes used for domestic switches and plugs. A picture of the current prototype implementation is shown in Figure 5. It is based on a previous device we have developed [33]. It has several I/O ports (the current version can handle up to seven digital outputs, one analog input, four digital inputs and four inputs that can be configurable as digital or analog) for the direct connection of simple devices, a custom Ethernet connector for the network, an expansion port with an I2C bus to connect complex devices that require their own memory and processing power, and an implementation of the generic gateway software for embedded devices, written in the C language, that is run on a 8-bit PIC 18F64J60 microcontroller.

An 8-bit microcontroller provides enough processing power to run an implementation of the UniDA distributed operation protocol and the control logic required to command many kinds of sensors and actuators. It also allows reducing the manufacturing costs of UniDA-D, as well as its power consumption. Likewise, if there is a device with processing or memory requirements that are higher than what UniDA-D provides, it can be deployed or connected to an expansion board with its own memory and processing capabilities, and interact with UniDA-D through the I2C bus of the expansion port. This last configuration may also be used to build new devices with the UniDA gateway integrated in their own hardware.

In order to control the devices that can be connected to the gateway, developers must write their own device drivers. These drivers are C modules that implement the logic to control a device, and fulfil a specific interface, similar to the *UniDA Device* and the *UniDA Device Operations Façade* of the generic gateway. These drivers can be added to the gateway software by editing a C file with their instantiation, and then programmed into a UniDA-D. Some default drivers are included in the default software, so every UniDA-D comes with preinstalled drivers for simple digital and analog input/output devices.

## 6. Practical Use Cases

The following section presents some practical use cases to illustrate some prominent characteristics of UniDA and compare it to other well-known technologies. The selected use cases are based on realistic situations that can happen in a typical HIE, a home. They have been extracted from some of the applications that were developed and deployed in the experimental setup constructed in our laboratory.

This experimental setup is deployed in four different rooms. Three of them are working areas and the other one is a meeting room. The setup is made up of a set of hardware devices and technologies that allow simulating and testing practical uses of common HIEs such as homes, offices or public environments. The main components of the experimental setup are:

- A KNX domotic BUS for lighting and central heating control, blind open/close operations, window/door state sensing or photo-cell sensing.
- A set of UniDA-D gateways with COTS hardware devices, like switches, motors, valves or temperature sensors connected to them.
- Some multimedia devices such as a media center device, a projector display and sound recording/playing hardware.
- A set of Android based Smartphones

Multiple applications and test examples have been developed and deployed in these experimental facilities. Among them we have selected three related scenarios that will be discussed in the remainder of this section to illustrate the characteristics and usage of the UniDA framework when solving realistic use cases.

The first scenario will be used to show the capabilities of the system for operation with multiple heterogeneous technologies, as well as to introduce the benefits of having a common and uniform model for device access. The second one builds up on the first scenario, enhancing it with new capabilities in order to show how easy it is not only to add new devices to a UniDA based application, but also to build and develop new devices compatible with it. Finally, the third scenario presents an example that highlights the benefits of a common model for device access enriched with semantic information. It displays the capability of a UniDA application for operation with very different types of devices in a uniform way.

**Figure 6.**
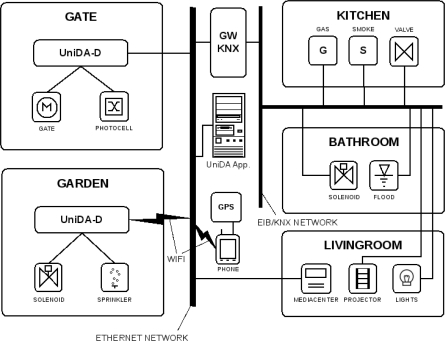
Test environment deployment including UniDA.

In order to make these scenarios more amenable, we have imagined a couple living in a home that is simulated in our experimental facilities with the deployment of a set of typical devices available in any modern home. This home has four different device subsystems that, without UniDA, operate in an isolated way: a domotic subsystem for lighting, blinds, the twilight switch, and security sensors such as smoke and flooding sensors; multimedia devices for entertainment; an automatic opening subsystem for the gate; and a garden irrigation system. As we do not have a garage door or an irrigation system in our laboratory, we have simulated them by means of motors and valves connected to two different UniDA-D devices. Figure 6 displays a schematic view of the test setup deployed in the virtual environment. This view includes some devices like a smartphone whose purpose will be described in the next subsections.

### 6.1. First Scenario

As indicated above, in this first scenario we are going to show how UniDA allows developers to easily build applications that can handle devices from multiple heterogeneous technologies without requiring any knowledge on the particular hardware involved. This characteristic allows developers to focus on what is important to them, the business logic of their application. It also makes the application directly compatible with a wide range of hardware technologies without requiring any extra effort.

For the first scenario, one of the subjects is arriving home in his car. He uses a remote to open the gate from the car and expects the system to turn on the garage and garden access path lights, as well as to turn off the irrigation system if it is powered up.

This example requires the use of three different subsystems in the home; the remote opening subsystem for the gate, the irrigation subsystem and the domotic subsystem for the lights. As these subsystems operate independently and with different technologies, some gateway devices must be used to build a UniDA network that can be accessed by applications using the UniDA library. The gate opening system was connected to a UniDA-D so it can be monitored by the applications. A UniDA-D was also used to control the irrigation system, and the KNX gateway, implemented using the generic gateway, was used to connect the domotic system to the network. A schematic of this installation, with the required gateways is shown in Figure 6.

To implement the desired functionality a device profile application was developed where the user can create profiles that associate the detection of a device state, for example, the activation of the gate photocell, to the execution of a series of commands in a set of devices, for instance, turn on the lights of the garage and the garden access path, and turn off the irrigation system. This application is implemented as a standalone Java application that uses the Jetty embedded HTTP server to offer two web GUIs, one for mobile devices and one for desktops. It stores the profile information in a HSQLDB database and uses the UniDA library to access the hardware devices associated to the profiles.

In this application the UniDA library was used for two different purposes. On one hand the common conceptual model was used to represent, manage and obtain semantic information about the devices and their capabilities. This way, the application can store and manage information from devices with very different technologies in a uniform way, without requiring the implementation and definition of different concepts for each device depending on its base technology and characteristics. On the other hand, the operational subsystem was used to discover the available devices, subscribe to their events and operate them in an homogeneous way, again without requiring specific logic for every technology involved, as the operational subsystem abstracts the underlying technologies behind the UniDA gateways, that translate the concepts managed by each particular technology to the common concepts managed by the UniDA framework.

### 6.2. Second Scenario

The second scenario builds on the first one with the aim of showing how new devices can be built and integrated in UniDA, as well as to highlight the benefits of using UniDA to support new devices, like direct support for the devices without requiring changes in the application code.

In this scenario, a second subject is arriving home in her car. She has a modern smartphone with GPS support, so she wants the system to make use of her location information to detect when she is arriving and automatically open the gate, turn on the access lights and turn off the irrigation system.

This example relies on all the hardware deployed for the first one, but requires the addition of user location sensing capabilities, for example the GPS sensor device of the user smartphone. Smartphones are often used by instrumentation systems as clients of their control applications, but their sensing capabilities are rarely exploited, even if they can provide really useful information about the users. Smartphones must operate in a distributed way, and they are very different devices in nature from the typical sensors and actuators available in current systems, so it is usually difficult and expensive to support their sensing capabilities.

**Figure 7.**
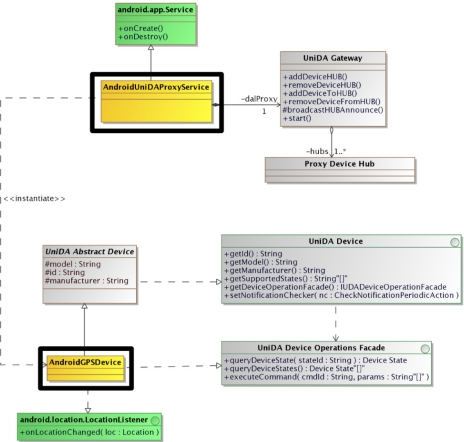
Class diagram of the UniDA gateway software for Android devices.

Thanks to the UniDA distributed design it is easy to integrate such distributed devices, being the easiest way to deploy a UniDA gateway in the device. For this particular use case, we have used the generic UniDA gateway component to build a gateway application for Android devices, so that any Android device could act as a device gateway for UniDA applications, providing access to its sensors and other devices. Figure 7 shows a class diagram of the UniDA gateway software for Android. As the Android SDK provides a Java API that is very similar to the J2SE API, it was only necessary to implement two new classes, marked with rectangles in Figure 7. The first one is a device driver for the GPS device (AndroidGPSDevice), and the second one is a wrapper to execute the gateway software as a background Android service (AndroidUniDAProxyService).

Depending on the capabilities of the new devices that are integrated in UniDA, they may be already supported by the DogOnt ontology. If they are supported, the library and its client applications would not need any modification at all. If the devices’ capabilities are not already supported by the ontology, as is the case of positioning devices like the GPS, it is not necessary to modify the UniDA library code, only the DOG ontology definition in the OWL language, by adding the new required concepts, which in this case are a location state and a positioning device. Furthermore, if the applications are coded generically enough, by relying on the semantic information provided by the ontology, they will be able to support entirely new types of devices without modifications.

With the gateway application for smartphone, UniDA based applications will be able to discover and use the GPS device. For example, with the device profile application, it will be possible to add a new activation condition to the profile defined in the previous use case that uses the GPS location information to know when the user is arriving home.

### 6.3. Third Scenario

This third scenario illustrates how the UniDA common conceptual model and uniform device operating paradigm facilitate the development of applications that cannot only use devices from multiple vendors and technologies simultaneously, but can contemplate very different kinds of devices using the same concepts.

In the third scenario, the couple has arrived at their home and want to watch a movie in their living room using their home cinema system. They want to use a remote they have for the domotic system to activate a ‘home cinema mode’ that changes the dimming of the living room lights, closes the blinds and turns on the video projector and the media center device.

To integrate the projector and the media center in UniDA, two different software gateways, which are run in an embedded PC, were developed. An UPnP gateway, which translates UPnP media renderer devices to UniDA, and a gateway that implements the PJLink protocol to remotely control video projectors.

UniDA applications can interact with any kind of hardware devices by using only a reduced set of common concepts thanks to the expressiveness of the UniDA common conceptual model and the DOG ontology.

Nevertheless, in order to support the wide array of functionalities available, these reduced set of concepts (devices, their associated states, control functionalities and commands) are enriched by metadata that specifies the particular states and control functionalities supported by each device. For example, this is the metadata associated to some devices involved in this scenario:

- The lights will be supported by the *DimmableLight* device concept of the DOG ontology. This concept includes an *OnOffState* and a *LightIntensityState*, as well as functionalities like *LightRegulationFuncionality* or *OnOffFunctionalitiy*, which include commands to power on/off the lights and change their intensity.
- The UPnP media renderer will be supported by the *MediaCenter* concept that includes *OnOffStandByState*, *VolumeLevelState* and *PlayState* states among others. As well as its associated control functionalities, like *PlayFuncionality*.

All these semantic metadata can be dynamically accessed from within UniDA client applications, so they can know the states available in each device and the commands to interact with them. Every device of the same type, for example, a media playing device, will have the same metadata independently of its implementation technology, and consequently an application can interact with every media playing device in the same way. Furthermore, multiple types of devices share some common functionality, like on/off or open/close, so it is even possible to control very different devices by using the same commands.

For this example, the previously described device profile application will be used again. The home cinema mode would be a profile defined to be activated by the state of a KNX device associated to a button of the remote. When the application receives a state change event about that button device, the application will issue the user specified commands to the corresponding devices. In this case an *OnCommand* command to the projector and media center devices, a *CloseCommand* to the blinds and a *StepDownCommand* to the lights. As can be seen, the application is only using different instances of those four concepts, and even for some interactions, it can use the same instances to operate really different devices coming from diverse hardware technologies.

## 7. HIE Instrumentation Technologies Comparison

Looking at the different HIE instrumentation solutions available it becomes clear that comparing them is not an easy goal. They come from very different fields and their original purposes were not the same, making it difficult to find a fair way to compare them. Notwithstanding the previous comments, here we will provide a comparison to the most prominent technologies available in order to clarify how UniDA stands against them.

The comparison process was started by determining a set of important issues that every HIE instrumentation technology should address. This set was based on our own experience in the development of solutions for HIEs and on a study of the current state of the art. Next, a set of key characteristics were extracted from those issues that will permit evaluating and comparing the different solutions available. Finally, the different approaches were characterized in terms of these characteristics and compared. In what follows we will describe each one of the characteristics selected and discuss how the different solutions perform in terms of those characteristics.

One of the main aspects that characterizes a HIE is the heterogeneity of the devices it can handle. There are few technologies that support many types of devices, and, consequently, it is an important feature for an HIE instrumentation system to include some kind of support to facilitate the use of diverse technologies. We have extracted three key characteristics to measure this aspect:

- *Integration of Heterogeneous technologies*. The capabilities of the solution to simultaneously use different hardware technologies.
- *Devices access paradigm*. How applications interact with the devices.
- *Supported devices*. The kind of devices that can be used.

HIE systems also require quite a lot information about the environment for their operation. This information, in its lower level, often comes from sensor and actuator devices deployed in the installation. As HIEs usually cover many physical places, devices must be physically scattered through the environment, thus, one important aspect can be how to remotely access and manage those devices. Two characteristics have been extracted to represent this aspect:

- *Distributed operation*. The ability of the technology to manage the distributed operation of the different elements of the solution.
- *Device discovery*. The ability of the technology to discover the hardware functionalities available in an installation.

In relation to this, to monitor and act over a physical environment, HIEs usually require quite a large number of devices. Therefore, the hardware required to deploy and manage these devices can be an important characteristic of the system, as it will critically affect the costs and power consumption bill of the installation. Two characteristics have been defined for this feature:

- *Hardware requirements*. A measurement of the hardware demand of the overall solution.
- *Instrumentation node*. Whether they include low power/cost hardware for the connection of devices to the system.

Finally, another important element of HIE instrumentation solutions can be the capabilities and tools that they provide to developers to facilitate the development and setup of HIE systems. From our perspective, this element has two main areas of interest:

- *Developer tools*. The capabilities that the solution provides developers to develop new HIE systems.
- *Interoperability*. The abilities of the solution to facilitate the utilization of software developed for other instrumentation technologies.

These nine characteristics may be taken as a good first approach in order to obtain a reasonable framework to compare device access solutions for HIEs, as they cover many important requirements of HIEs’ developers, installers and users.

In addition to establishing this set of features for the comparison of device access solutions for human interaction environments, we have also performed a selection of relevant projects and technologies that provide solutions in this area. They are six well know solutions selected due to their relevance in the market or the research community. Table 1 shows a comparison of these technologies and UniDA with respect to the previously defined nine characteristics.

AMIGO is one of the biggest and more advanced projects in the realm of Ambient Intelligence, a field typically related to HIEs. As such, they have developed some interesting solutions in the area of device interaction. AMIGO proposes UPnP technology as its main device access solution, but it uses proxy components to encapsulate devices and hardware technologies, existing prebuilt proxies for some well-known domotic technologies, in addition to UPnP. These features allow AMIGO to be directly compatible with some of the most generally used technologies nowadays: UPnP for multimedia and domotic technologies for building automation. Nevertheless, it relies on UPnP standard interfaces for device interaction, and it does not share common interfaces among every type of device, as a consequence applications require specific logic and knowledge to interact with different devices.

AmI-Space is another integral solution for Ambient Intelligence systems that has implied the development of some specific technologies for HIE instrumentation. It proposes a proxy-like component to connect the devices to the AmI system, and its creators have built a hardware proxy device that can be used to build new devices directly compatible with AmI-Space or to connect legacy devices. This way, their hardware requirements are lower than many other solutions, especially when using simple or legacy devices. The biggest drawback is that, as AMIGO, it does not contemplate a common interface for device interaction.

DOG, Domotic OSGi Gateway, is a solution for building automation, specially focused on domotic technology integration. The most important feature of DOG is the use of an ontology to model the instrumentation network. This allows DOG to share a common device interaction interface across all different types of devices, as well as to provide some advanced device management capabilities thanks to the inheritance properties supported by the ontology. Nevertheless, the main drawbacks of DOG are that it only supports domotic technologies and its inability to operate in a distributed manner.

Domotic technologies are the most widely used solutions for building automation. They are easy to find in the market and are even included sometimes in the building electric installation. These technologies typically use peer-to-peer approaches, so they work very well in a distributed fashion, and they use low power hardware to interconnect the devices. The most important drawbacks are that they often require specialized network setups; they have a quite small variety of devices and do not include any capability to interoperate with other technologies.

UPnP is, together with domotic technologies, one of the most widely commercially supported technologies for device access. Although it is designed to be a general device access solution, the market reality is that it is being used almost exclusively for multimedia devices, especially multimedia streaming devices. UPnP uses a service based approach for device technology encapsulation, providing standard interfaces for many kinds of devices. The main problems of UPnP are that it does not share a common paradigm among the interfaces for each kind of device, so applications that use different types of devices must contain logic to support each one of them. Furthermore its hardware requirements are medium, especially when talking about simple domotic devices.

SAIL is another solution that comes from the Ambient Intelligence field, as it is highly related to the PERSONA project. SAIL shares many similarities with DOG; as both of them do not support distributed operation, both have medium hardware requirements, as they must be run in embedded PCs or similar devices, and both of them provide a uniform device access paradigm. Therefore, it shares the problems of DOG plus another one, SAIL was originally designed to allow the interaction with Wireless Sensor Networks, so currently it does not support any other kind of devices. Additionally SAIL performs well in the interoperability features, as its devices can be exported as UPnP devices, making them usable from UPnP compatible applications.

Finally, UniDA is specially designed for HIE environments, as such, it has good distributed operation support and low hardware requirements, as it includes an instrumentation node that can be used to connect sensor/actuator devices. It includes capabilities for the integration of other existing technologies so that systems using UniDA can take advantage of existing installations. It also shares a uniform device interaction paradigm among all kind of devices, thus facilitating the development of applications that require the use of different devices. The main drawback of UniDA is its current low interoperability capability, as, although it is very easy to use any other technology from UniDA, it cannot be directly used by systems developed using other technologies, such as for example SAIL does with UPnP, or AMIGO with OSGi, as it does not provide direct external interfaces for other technologies.

Bearing in mind the different pros and cons of each technology, it can be concluded that technologies like DOG and SAIL may be good candidates for small sized systems that do not require distributed operation, being SAIL especially interesting when interaction with WSNs is required. Domotic technologies, on the other hand, are good candidates for systems with simple requirements, as they are already available in the market, but they do not support many advanced functionalities. UPnP is a good solution for multimedia because it is almost a *de facto* standard for those kind of devices. AMIGO and AmI-Space can be good options for complex systems as they support distributed operation and complex devices, they will work especially well if many different kinds of devices are not required. Finally UniDA can be a very good option for systems that require a lot of different types of devices, or that need to use a mix of legacy and new devices. It can also be a good candidate for systems with highly distributed operation requirements.

## 8. Conclusions

In the last few years a clear trend in consumer electronics towards a continuous increase in the integration of devices in the daily lives of people has been observed. This integration is making it possible to build human interaction environments following the visionary ideas of Ubiquitous Computing and Ambient Intelligence. In accordance with this trend, some technologies have proliferated providing functionalities such as interoperability for multimedia devices, basic environment sensing with domotic technologies, or synchronization between different devices. Nevertheless, they fall short in the support and integration of the variety of devices and technologies required by HIE systems. The academic community has identified some of these shortcomings and numerous research projects, such as DOG, AMIGO or PERSONA, have been developing solutions in the area.

Each of those projects comes from a different background, and they are not specifically focused on HIE systems, so, even though they solve some of the issues associated to HIEs, they do not solve them completely or in an integrated way. As we have shown in the comparison of Section 7, some of the major gaps are identified in the support for distributed operation, the lack of a uniform device access paradigm and the relatively high hardware requirements. In the last few years we have been developing the UniDA framework, an integral solution for HIE systems that addresses those specific problems. It provides developers and installers with a conceptual framework capable of modelling an HIE together with a set of libraries, tools and devices that allow the building of low cost distributed instrumentation networks with support for transparent integration of other technologies.

In this paper, the UniDA framework has been described from two different points of view. From a conceptual perspective the framework has three components: A common conceptual model for the description of an instrumentation network, a uniform paradigm for device access and a distributed operation protocol for the interaction between the different distributed elements of the system. From an implementation perspective it is designed as a distributed system made up of an implementation of the model and uniform device access paradigm as a Java library, a set of software and hardware gateway components that make up an instrumentation network and a distributed operation protocol to allow the communication between the library and the devices. As a result of testing the proposed solution in a series of realistic use cases we have extracted some of the benefits of using the UniDA framework for the development of HIE systems:

- Having a uniform conceptual framework for device access allows developers to ignore the details of the different devices, protocols, *etc.*, deployed on an installation; allows applications to be used in different installations with varying devices without requiring changes; and allows the user to change the devices without requiring reconfiguration or changes in the system.
- The distributed architecture of UniDA facilitates the integration of many kinds of devices and technologies in an HIE.
- The UniDA-D instrumentation node facilitates building distributed instrumentation networks with COTS hardware and low power consumption requirements.

Nevertheless, some future work is already required to achieve a complete solution for HIEs. The current implementation of the distributed operation protocol is quite limited; some efforts must be made to make it more efficient and reliable. Another important area of improvement is the autonomy of the system. The current implementation delegates all the autonomy in the clients, providing only devices access capabilities. Our main future goal is to provide UniDA gateways with autonomy, so the clients can delegate some control responsibilities directly to the instrumentation network, and operate exclusively as monitors of the network.

## Figures and Tables

**Table 1.** Comparison table of instrumentation technologies for HIEs.

	AMIGO	AmI-Space	DOG	Domotic	UniDA	UPnP	SAIL

Integration of Heterogeneous technologies	Yes	Yes	Yes	No	Yes	No	Yes
Device access paradigm	*Ad-hoc*	*Ad-hoc*	Uniform	*Ad-hoc*	Uniform	Device specific	Uniform
Device discovery	Yes	Yes	No	Infrequently	Yes	Yes	No
Distributed operation	Medium	High	Low	High	High	Medium	Low
Hardware requirements	High	Medium	Medium	Low	Low	Medium	Medium
Supported devices	Domotic, UPnP	COTS, UPnP	Domotic	Domotic	COTS, Domotic, UPnP	UPnP	WSN
Instrumentation node	No	Yes	No	Yes	Yes	No	No
Developer tools	OSGi services	Agent-based middleware	Ontology, XML-RPC API	Propietary APIs	Ontology, API, WS, device development, tech. integration	APIs for multiple languages	Exported as UPnP device
Interoperability	Medium	Low	Low	No	Low	Medium	Medium
